# A decision-making model for public health authorities in circumstances of potentially high public risk

**DOI:** 10.1093/pubmed/fdaf052

**Published:** 2025-05-18

**Authors:** Fatima N Dalal, Simon E Kolstoe, Yimmy Y Chow, Dipti Dashore, Marc Lipman, Patrick Lillie, Simon Padfield, Roger Gajraj, Carmel McGrath, Tom Fowler, Susan L Ibbotson

**Affiliations:** UK Health Security Agency, 10 South Colonnade, London E14 5EA, UK; UK Health Security Agency, 10 South Colonnade, London E14 5EA, UK; School of Dental, Health and Care Professions, University of Portsmouth, St Andrews Court, Portsmouth PO1 2UP, UK; UK Health Security Agency, 10 South Colonnade, London E14 5EA, UK; UK Health Security Agency, 10 South Colonnade, London E14 5EA, UK; Royal Free Campus University College London, Rowland Hill Street, London NW3 2PF, UK; Royal Free London NHS Foundation Trust, Pond Street, London NW3 2QG, UK; Hull University Teaching Hospitals NHS Trust, Hull Royal Infirmary, Anlaby Road, Hull HU3 2JZ, UK; UK Health Security Agency, 10 South Colonnade, London E14 5EA, UK; UK Health Security Agency, 10 South Colonnade, London E14 5EA, UK; NIHR Health Protection Research Unit in Behavioural Science and Evaluation, University of Bristol, Bristol, UK; The National Institute for Health and Care Research Applied Research Collaboration West, University Hospitals Bristol and Weston NHS Foundation Trust, UK; Faculty of Health and Applied Sciences, School of Health and Social Wellbeing, University of West England, Bristol, UK; UK Health Security Agency, 10 South Colonnade, London E14 5EA, UK; Queen Mary University of London William Harvey Research Institute, Queen Mary University of London, Charterhouse Square, London EC1M 6BQ, UK; UK Health Security Agency, 10 South Colonnade, London E14 5EA, UK

**Keywords:** ethics, infectious disease, public health

## Abstract

**Background:**

An expert multidisciplinary panel was commissioned by a UK Health Security Agency led incident management team (IMT) to support decision making in the case of an individual with extensively drug-resistant tuberculosis. The behaviour and stated intentions of the individual were potentially a significant risk to public health, and the regional IMT felt unable to adequately balance the rights of the individual, versus the public health risk, within current processes and legal powers.

**Method:**

We describe the composition, organization, implementation, and conclusions of a national, expert, multidisciplinary panel.

**Results:**

The national panel convened over three structured virtual meetings to consider the balance between the rights of the individual to an unrestricted life, and the duty to protect the public’s health. Evidence included briefs from the regional IMT and input from a public consultation group. Following the first two meetings the need for a literature review examining the success of surgical interventions was identified and conducted.

**Conclusions:**

Evidence and conclusions were mapped onto a custom-designed risk assessment template. The panel provided authoritative advice regarding the case, and developed a review methodology that is transferable to similar complex public health scenarios both in the UK and internationally.

## Introduction

Complex infectious disease cases contribute significantly to the workload of public health systems, particularly clinicians who are involved in the care of the individuals, and public health staff leading incident management teams (IMTs). IMTs are a common construct used within the UK to coordinate health protection responses where cross-organizational working is required. In some cases, despite exploring and implementing relevant clinical and treatment options, the ongoing public health risk cannot be adequately reduced through standard IMT approaches. This is either because the IMT does not have the relevant ethical and legal expertise (or powers) or if there is a perceived need for greater objectivity (as members of the IMT are often also involved with the clinical care). While the ultimate responsibility for actions taken lies with the IMT, accessing alternative sources of advice and information, including public consultation, may be a solution to enable and demonstrate relevant diligence in decision making.^1^

This was the situation for the IMT leads in a 2024 complex case of extensively drug-resistant tuberculosis (XDRTB) in a highly populated metropolitan area in the UK. Under time–pressure to give a longer-term management decision, they commissioned national colleagues at the UK Health Security Agency (UKHSA) to facilitate an external expert panel that could provide an approach, and suggest interventions, that would balance the rights of the individual to an unrestricted life with the need to protect the public’s health. At the time of commission, the individual was a hospital inpatient under a health protection part 2A order. Part 2A orders are granted when local authorities ‘*need additional powers to manage a person or item that may cause significant harm to human health from infection or contamination*’.^2^

In this paper, we outline the steps undertaken to set up the expert panel, the learning and outcomes from the panel, and the lessons identified through an after-action review of the process. We present a method that can be used in other, similar, situations.

## Method

A project group was initially created within UKHSA composed of the Head of Clinical Excellence and Quality (to subsequently serve as panel chair), the Deputy Director of Regional Operations, a Clinical Fellow, and administrative support, to develop the approach for the expert panel’s membership and inputs.

A literature search using PubMed, Ovid Medline, and Google Scholar, using the key terms ‘tuberculosis’, ‘expert panel’, ‘public health’, and relevant synonyms in a variety of AND/OR combinations did not identify reports of similar panels or methodologies in the UK or internationally, although the search was limited to English language sources (Supplementary Data 1).

The project group met frequently over 6 weeks to establish panel membership, develop the Terms of Reference, and identify legal and behavioural science expertise. The chair also reviewed the Code of Practice for Scientific Advisory Committees^3^ to establish ways of working.

A Delphi^4^ approach was initially considered but this was considered incompatible with the short timescale required for the advice. However, a consensus-forming, iterative, principle was incorporated into the methodology.

The question posed by the IMT lent itself to structuring the conversation around the risks to the individual and the wider public, and options for mitigation in several different scenarios ranging from: ‘the patient is not infectious and is compliant with treatment and advice’ to ‘the patient is infectious and non-compliant’ (see results).

As part of the preparatory work the project group developed:

- A panel Terms of Reference including desired members (Supplementary Data 2). This included a confidentiality statement for panel members and indemnity protection assurance.
- An overview of the case and potential issues to be addressed produced by the IMT chairs.
- An initial draft of risk levels and a proposed assessment template for use during the panel discussion was formulated, with the latter based on a scenario structure suited to the IMT’s request.
- Materials to facilitate a small public consultation group supported by the internal UKHSA Behavioural Science Insights Unit (BSIU) (Supplementary Data 3).

Once the panel had been formed, three virtual meetings were held approximately 1 week apart. The public consultation group exercise, with six public participants, was conducted between the first and second panel meetings.

## Results

### Membership

Using networks and relationships within the UKHSA, membership was as described in Table 1.

**Table 1:** Panel membership.

A consultant in public health with clinical governance expertise and senior leadership experience of responding to complex TB cases (chair of panel) Consultant respiratory physicians with expertise in the management of drug-resistant TB (including the clinician caring for the patient and two clinicians from another region with extensive experience of caring for patients with relatively rare XDRTB) A consultant microbiologist with specialist TB laboratory expertise (from one of the devolved nations A consultant in communicable disease control from another region with significant experience in Public Health aspects of care of patients with drug-resistant TB A director of public health with experience in the community and public health management of people with drug-resistant TB A lay panel member with personal lived experience of TB. Lived experience was considered a particularly important form of expert input and advocacy. A member of the project team met with the lay panel member prior to the first panel meeting to ensure they felt updated and facilitated to contribute freely A professor of bioethics to bring an ethical and human rights perspective to the panel’s deliberations, and ensure that the panel observed good research governance, as required A senior public health practitioner with Caldicott Guardian expertise and experience to ensure the panel observed good practise in line with Caldicott requirements UKHSA’s General Counsel bringing expertise and experience of the public health legal aspects of managing infectious diseases The IMT chairs—bringing their knowledge of the IMT’s discussions and the local situation. As this was an expert panel and not an independent panel, there was no conflict in including these members on the panel, indeed their knowledge of the case was essential to ensure that the panel was as well informed as possible about the circumstances of the case Observers including NHS England specialized commissioners and the academic lead for an independent evaluation of the expert panel’s method

### Panel meetings

#### First panel meeting

**Figure 1:**
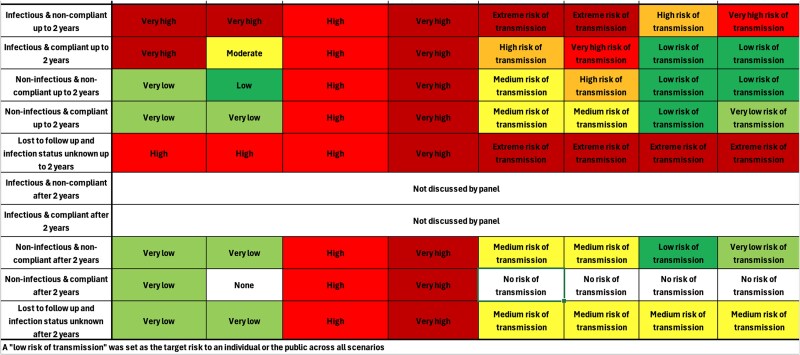
The completed risk assessment matrix.

The first meeting covered the agreement of the Terms of Reference (Supplementary Data 2), introducing panel members to the characteristics of the case and potential scenarios, and an explanation of a proposed risk assessment methodology. This included the proposed table to help identify risk levels (see Fig. 1). During this meeting, updates to the risk assessment methods were discussed. A number of legal questions were also identified and put to the UKHSA’s legal counsel who was a member of the panel.

#### Second panel meeting

The second meeting considered the output from the public consultation group. A need to consider additional evidence on the likelihood of cure (at 2 years) following lobectomy for localized XDRTB disease was identified. A clinical panel member (Consultant Respiratory Physician) agreed to conduct this rapid literature review prior to the third meeting.

‘In committee’ discussions began the population of the risk assessment tool. Although completion ‘in committee’ was anticipated to be too lengthy, for time-efficiency these discussions set the direction and principles, which were taken forward into a sub-meeting between a project group member and the IMT chairs to draft a completed matrix ready for the final panel meeting. The draft completed matrix was circulated to all panel members prior to the final panel meeting.

#### 3^rd^ panel meeting

This meeting considered the draft completed risk matrix (which was updated ‘live’ following any challenge/endorsement from panel members), alongside the clinical evidence on surgery to treat extensively drug resistant (XDR) or multi-drug resistant (MDR) pulmonary tuberculosis (TB) (Supplementary Data 4). Following discussion, the risk matrix was finalized and a list of consensus statements drafted.

### Summary of panel inputs

(a) The public consultation group report (presented at Meeting 2; Supplementary Data 3).
  This was conducted through collaboration with the UKHSA’s BSIU team, and their Patient and Public Involvement and Engagement network which included the Behavioural Science and Evaluation Health Protection Research Unit at the University of the West of England (UWE). It was held virtually for a single one and a half hour meeting on a weekday evening. Six public participants attended, identified through local advertisement. Payments of £25/hour were provided to each participant. Prior to the meeting, participants were provided with pre-reading, that while containing no case specific information, did introduce the relevant issues collated using publicly available resources on Tbalert.org.^5^ The meeting was facilitated by two project members and a member of the Health Protection and Research Unit in Behavioural Science and Evaluation.
  The narrative report from the public consultation meeting described view on scenario-based questions that included an individual with XDRTB being: (i) compliant with treatment and non-infectious, (ii) non-compliant and infectious, and (iii) non-compliant with unknown infection status. The group agreed with a risk-based approach and acknowledged the importance of balancing the needs of the individual with the need to protect public health. They felt that risk of transmission should be managed to be ‘as low as reasonably possible’ while, in the scenario of compliant with treatment, enabling the individual to live as normal a life as possible. They also understood that risk of transmission could not be negated entirely. In the scenario of a patient being infectious and/or non-compliant, the group expected the authorities to use legal powers to keep the public safe. When the expert panel discussed the public consultation report, panel members noted that the findings of a single public consultation group discussion was not representative of all public views on the issue. However, the purpose of the public consultation group was to provide wider individual representation to the panel.
(b) Summary of the clinical evidence on surgery to treat XDR/MDR pulmonary TB (Supplementary Data 4).
(c) A document created by UKHSA General Counsel answering the legal questions posed by the IMT.

### Panel outputs 1—the risk assessment matrix and risk tables

The aim of creating a risk assessment matrix was to quantify the probability, impact, and risk of transmission in a number of different scenarios, while also defining the target position following mitigation actions.

Following agreement on the risk levels and structure of the risk assessment matrix, the panel identified (a) risk levels for the five scenarios (following the second panel meeting, the scenarios were expanded to include the risk at 2 years following definitive surgical treatment), (b) interventions to reduce risk, and (c) assumptions and rationale for the assessment.

#### Risk levels and scenarios

During the first meeting, the panel decided that the complexity of the case and proposed scenarios justified six levels of risk rather than the initially intended three. It was also noted that risk levels were relative in lieu of any agreed method to precisely quantify risks. The panel agreed that the overall target was to reduce the residual risk to ‘as low as reasonably practical’ rather than ‘none’, again due to difficulties in precisely quantifying risk. This approach was supported by the output of the public consultation group as discussed by the panel in the second meeting.

**Figure 2:**
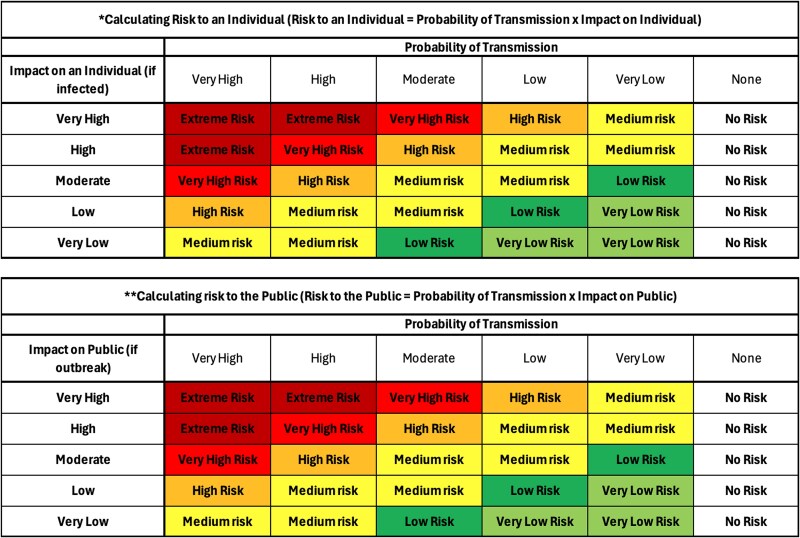
Risk calculation tables (for Columns 5 and 6 of the risk matrix).

When considering the risk to both an individual and the public, risk was quantified as a product of likelihood of transmission (‘Probability of Transmission’) and the impact of infection (‘Impact on Individual/Public’). The tables shown in Fig. 2 were developed, and these were used to populate Columns 5 and 6 in the overall risk assessment.

During the first panel meeting the five scenarios (infectious and compliant, infectious and non-compliant, non-infectious and compliant, non-infectious and non-compliant, lost to follow-up infection unknown) were expanded to include risk up to 2 years, and after 2 years. This was because the expert clinical opinion, supported by the available published literature (Supplementary Data 4), allowed the panel to conclude that 2 years was an appropriate timeframe to discriminate between infectious and non-infectious following lobectomy.

#### Interventions to reduce risk

##### Clinical treatment options

As the disease strain was resistant to all antimicrobials in the standard treatment regimen, and due to the individual’s history of non-compliance, the panel understood that no further drug treatment was clinically advised. During the first panel meeting, the clinical effect of lobectomy was discussed, with clinical members advising anecdotally that if patients were infection free after 2 years they could be considered ‘cured’. To confirm this, evidence from a rapid literature review was requested. This evidence supported by the World Health Organization’s consolidated guidelines on tuberculosis. Module 4^6^ was particularly valuable as following the discussion the panel were optimistic that a successful outcome both for the individual and the public health was possible.

Mental health support was also discussed, although this would only be possible for the scenarios where the patient was compliant (see below).

##### Long-term engagement

Given the current lack of suitable facilities (long-term negative pressure room availability outside of an acute hospital setting) and health protection legal powers within the UK, the panel considered that effective long-term engagement of the individual was the most effective mechanism to reduce the risk of transmission. Legal powers would only be relevant in a scenario of non-compliance, and the panel recognized that UK legal powers were limited and may be operationally unenforceable. This made effective engagement the most potent mechanism to address the high risk of transmission.

The IMT had concerns regarding the individual’s foreign travel which would raise further monitoring challenge and the legal advice provided to panel members outlined no alternative to engagement. Given the very high public health risk were the patient to be lost to follow up and/or infectious, the panel recommended that the partners in the IMT do as much as possible to ensure the patient’s ongoing engagement with services. In the scenario of no fixed abode, and the individual moving from area to area, the panel discussed the importance of a single lead local authority (LA) to coordinate wider support for the individual irrespective of whereabouts. Similarly, maintaining continuity of contact with NHS TB services and a named lead clinician was important.

Other opportunities for long-term engagement included peer support for substance use, and ongoing monitoring/engagement from primary care/general practice (GP) services. Again, these options would only be available should the patient be compliant (see below).

##### Comprehensive support package

A comprehensive support package could be recommended covering aspects including access to job planning services, financial benefits, suitable accommodation; the consideration of financial, technology-based, or other incentives; continuity of care from a GP practice; peer and other support for substance misuse; and mental health support with ongoing coordination of the wider support package.

##### Further legal interventions

The main option was the use of The Health Protection (Part 2A Orders) Regulations 2010. The necessity of a part 2A order is determined by a magistrate assessing the evidence provided by the LA. There is no limit to the number of times an application can be made. Whether it is granted is determined by the evidence.

The panel also noted Article 8 of the European Convention on Human Rights; the right to a private life.^7^ This is a qualified right as public authorities can interfere with an individual’s right to respect for private and family life, home, and correspondence, in certain situations if they show their actions are lawful, necessary, and proportionate so as to protect public safety and health.

In the scenarios of non-compliance, given the associated risk to public health, further application(s) for an appropriate Part 2A order were recommended as necessary and proportionate, while recognizing a Part 2A order may be of limited effectiveness unless the individual could be adequately supported to adhere to the order.

If an individual breaches a Part 2A order, the only sanction is a fine which may be meaningless if the individual lacks the means to pay. Police power to enforce a Part 2A order is limited to taking the individual into custody and returning the individual to the place of detention, isolation, or quarantining as set out in the Part 2A order. Prosecution for reckless transmission, may be relevant in the event of transmission, but has not been tested in the English courts for cases of tuberculosis. Individuals have been prosecuted with custodial sentences in cases of reckless transmission of human immunodeficiency virus (HIV), which hinged on the whether the newly infected person had consented to the risk of HIV infection.^8^ The respiratory route of transmission of tuberculosis does not lend itself to consent of risk of infection and a precedence has not been set.

World Health Organization guidance^6^ states that patients with confirmed infectious or potentially infectious pulmonary TB should be advised not to travel on commercial aircraft until there is no longer a risk of transmitting infection to others. If travel is imperative then a travel protocol should be agreed on between the patient, the local public health authority and the airline in question, with the risk of infection of passengers with MDR/XDRTB assessed using national guidelines. This guidance clearly assumes a scenario of compliance of the individual with the clinical and public health team.

The International Health Regulations^9^ would require that UKHSA notify the appropriate public health authority in another country if they were aware that the individual with infectious TB was going to travel, against this advice. The relevant airline could potentially also be informed. Clearly this would only be possible if the individual continues to engage with the authorities. For TB cases, there are no public health legal powers that would enable UKHSA/LAs to prevent the individual from boarding a flight or a ship.

### Panel outputs 2—the expert panel report

Following the conclusion of the panel meetings, an expert panel report was drafted. All panel members agreed, and signed off, the final report with no abstentions. The report was submitted to the IMT as a consensus statement from the panel.

## Discussion

### Main findings

Throughout all three meetings, members commented that capturing and reporting issues raised during the panel discussions would benefit the handling of future cases. A subsequent independent ‘After Action Review (AAR)’ was conducted to record learning because it was noted that while such activities are often recorded internally, they are seldom shared with the wider community to assist the handling of similar cases or situations.

The methodology and inputs, including the views of the public consultation group, therefore provide an example of a successful approach to a public health scenarios where the rights of the individual were balanced with the need to protect the public.

### What is already known on this topic?

The initial literature found no published reports of similar panels or methodologies described nationally or internationally in the English language. The panel highlighted the lack of legal powers and facilities to support someone to comply with follow-up over a lengthy period, with this information known by the professional community prior to the panel convening. This is an obvious challenge to reliably protecting the public’s health in this kind of scenario, and it is of interest that the public consultation group expected that the health authorities could, and would, use powers defined in law. They were thus unaware of the absence of effective legal powers.

### What does this publication add?

This paper provides a template methodology and resources for colleagues who may face similar public health scenarios in the future. Our experience described here may help others to identify the resources required, costs involved, and further develop a framework for scenario-based risk assessment. Conclusions from the ARR included the process was time and resource-heavy, with the set up and running of the panel requiring significant project management and leadership. Thus said the financial costs were low with the only direct cost being payment of £25/hour to each of the public participation group members (as per established National Institute for Health Research practice £225 in total). Time spent by UKHSA staff was away from their day-to-day roles and while other external panel members gave their time for free, this is likely to have had a similar opportunity cost.

The AAR highlighted the recognition the non-UKHSA panel members had for the importance of the panel; both on the subject matter and the multi-disciplinarity and multi-professionalism of the members.

### Limitations

The time-constraints on the project were keenly felt due to the 6–8 weeks within which a decision was needed due to the IMT’s deadline, which also meant that more extensive public consultations were not possible. It was also of note that no separate ethics review was conducted for the work described here, albeit an experienced bioethicist and research ethics committee chair was included on the panel. An alternative may have been to also seek the views of a clinical ethics committee, however, along with adding further time to the process, the lack of an explicit ethics committee review is not uncommon for both the collection of public views (e.g. UK’s Health Research Authorities guidance on Public and Patient Involvement^10^), while clinical and public health advice almost always falls outside the usual remit of research ethics regulators^11^ and committees. Had time allowed, a more systematically robust approach could have been implemented such as the Delphi method^4^; however, our aim from sharing our methodology of this time-pressured case was to provide a process and starting point that can be developed and adapted by future users.

## Conclusion

The approach described in this paper could be beneficial in similar operational scenarios where ethical decisions balancing the rights of the individual with the need to protect the public’s health are made regularly without a framework, and also in the context of operationally unenforceable legal powers. Often these decisions need to made rapidly using best available evidence and experience.

The findings are relevant to future cases of XDRTB but may be widely applicable to other complex infectious diseases including those designated as high consequence infectious diseases (HCIDs). A template risk assessment matrix is included for use by others in such cases (Supplementary Data 5).

## Supplementary Material

Supplementary_Data_1_XDRTB_Expert_Multidisciplinary_Panel-Literature_Search_fdaf052

Supplementary_Data_2_XDRTB_Expert_Multidisciplinary_Panel-Terms_of_Reference_(1)_fdaf052

Supplementary_Data_3_XDRTB_Expert_Background_Info_fdaf052

Supplementary_Data_4_XDRTB_Expert_Multidisciplinary_TB_Evidence_Summary_fdaf052

Supplementary_Data_5_Expert_Multidisciplinary_Panel-Template_Risk_Assessment_Matrix_fdaf052

## Data Availability

All data that can be shared publicly without compromising patient confidentiality has been incorporated into the article and the supplementary materials. There is some data that cannot be shared publicly to ensure patient confidentiality.

## Notes

**Fatima N. Dalal,** Clinical Fellow

**Simon E. Kolstoe,** Associate Professor of Bioethics

**Yimmy Y. Chow,** Deputy Director and Consultant in Health Protection

**Dipti Dashore,** General Counsel

**Marc Lipman,** Consultant in Respiratory & HIV Medicine

**Patrick Lillie,** Consultant in Infectious Diseases

**Simon Padfield,** Consultant in Health Protection

**Roger Gajraj,** Consultant in Health Protection

**Carmel McGrath,** Senior Research Fellow in Public Involvement

**Tom Fowler,** Director: Public Health Testing

**Susan L. Ibbotson,** Head of Clinical Excellence and Quality
