# Supplementary material for: A decision-making model for public health authorities in circumstances of potentially high public risk
Source: J Public Health (Oxf). 2025 May 18;47(3):550–7. doi: 10.1093/pubmed/fdaf052 (PMC12395956; doi:10.1093/pubmed/fdaf052)
Supplement: Supplementary_Data_1_XDRTB_Expert_Multidisciplinary_Panel-Literature_Search_fdaf052 [file supplementary_data_1_xdrtb_expert_multidisciplinary_panel-literature_search_fdaf052.pdf]

Literature Search – What/is there an evidence base for independent public health advisory panels in cases of tuberculosis?

22/11/2023

Keywords from question posed:

- Public health
  - Synonyms: community health
- Advisory panel
  - Synonyms: advisory board, advisors, consultative board, consultants, board of advisors, expert committee, committee, panel, council, board of experts, expert panel
- Tuberculosis
  - Synonyms: TB, consumption, phthisis, extensively drug-resistant tuberculosis, XDR-TB, extensively drug resistant tuberculosis, XDRTB, drug resistant tuberculosis, drug-resistant TB, drug resistant TB, DRTB, multi-drug resistant tuberculosis, MDRTB, MDR-TB,
  - OR: chronic infection, long term infection, long-term infection, infectious disease

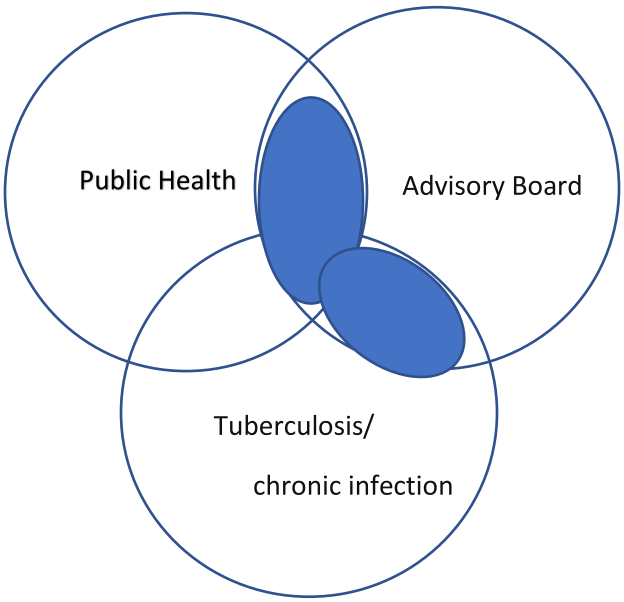

Keywords also used in search:

- Ethic* issues
- Ethic* challenge*

Databases searched:

- ***PubMed*** – main focus
- Ovid Medline
- Google Scholar

- Google

Summary of literature search findings:

- **There were no sources identified in the literature search that referred to an independent public health advisory panel in individual cases of tuberculosis**
- **The literature search identified several peer-reviewed articles, international, national and regional guidelines that emphasized the importance of community-based public health management at an individual case level, balancing public health risk with international human rights.**

Top 10 relevant search findings (ethical focus highlighted in grey):

Article/Source	Database Searched	Date Searched	Relevance
Matteelli A, Centis R, D'Ambrosio L, Sotgiu G, Tadolini M, Pontali E, Spanevello A, Migliori GB. WHO strategies for the programmatic management of drug-resistant tuberculosis. Expert Rev Respir Med. 2016 Sep;10(9):991-1002. doi: 10.1080/17476348.2016.1199278. Epub 2016 Jun 22. PMID: 27276361.	PubMed	22/11/2023	“it is mandatory to tackle the social determinants and socio-economic barriers favouring the MDR-TB”
Taylor Z, Nolan CM, Blumberg HM; American Thoracic Society; Centers for Disease Control and Prevention; Infectious Diseases Society of America. Controlling tuberculosis in the United States. Recommendations from the American Thoracic Society, CDC, and the Infectious Diseases Society of America. MMWR Recomm Rep. 2005 Nov 4;54(RR-12):1-81. Erratum in: MMWR Morb Mortal Wkly Rep. 2005 Nov 18;54(45):1161. PMID: 16267499.	PubMed	22/11/2023	“ When serving a population at risk for TB, community-based organizations should become involved in advocacy initiatives, such as state and local TB advisory committees and coalitions”
Cole B, Nilsen DM, Will L, Etkind SC, Burgos M, Chorba T. Essential Components of a Public Health Tuberculosis Prevention, Control, and Elimination Program: Recommendations of the Advisory Council for the Elimination of Tuberculosis and the National Tuberculosis Controllers Association. MMWR Recomm Rep. 2020 Jul 31;69(7):1-27. doi:	PubMed	22/11/2023	“TB control programs should develop an overall TB control strategy in collaboration with local partners (e.g., health care providers, professional societies, and voluntary organizations) and state, local, and tribal advisory committees,”

10.15585/mmwr.rr6907a1. PMID: 32730235; PMCID: PMC7392523.			
Collaborative TB Strategy for England 2015-2020. Public Health England. Published March 2021. Collaborative TB Strategy for England, 2015 to 2020: end of programme report (publishing.service.gov.uk)	Google	22/11/2023	"provide advice and support to clinicians caring for TB patients with MDR-TB and complex TB particularly those requiring Bedaquiline and Delamanid. Offer monthly virtual MDTs where MDR-TB cases are discussed by those experienced in the care of patients with MDR-TB"
Strategic and Technical Advisory Group for Tuberculosis (STAG-TB) – World Health Organisation	Google	22/11/2023	"STAG-TB is comprised of 15 eminent experts from ministries of health, national TB programmes, academic and research institutions, civil society organizations, and communities and patients affected by TB" "The mission of the STAG-TB is to contribute to ending the tuberculosis epidemic, and eventually eliminating the disease, by providing state-of-the-art scientific and technical guidance to WHO"
Lange C, Dheda K, Chesov D, Mandalakas AM, Udwadia Z, Horsburgh CR Jr. Management of drug-resistant tuberculosis. Lancet. 2019 Sep 14;394(10202):953-966. doi: 10.1016/S0140-6736(19)31882-3. PMID: 31526739.	PubMed	22/11/2023	"Care should be patient focused and oriented (the first pillar of WHO's End TB strategy to end tuberculosis), encompassing patient choice, and be empowering, dignified, and respectful.Patients who cannot be treated successfully with medical treatment alone, or who terminate medical treatment, should have access to surgical intervention and palliative care if appropriate. Multidisciplinary, long-term, community-based residential facilities should be available to cater for patients who cannot be cured and who cannot be managed at home."
Provincial Infectious Diseases Advisory Committee on Infection Prevention and	Google	22/11/2023	"The committee advises Public Health Ontario on the

Control (PIDAC-IPC) - Public Health Ontario			prevention and control of health care-associated infections, considering the entire health care system, with a focus on clients, patients and residents, as well as health care providers"
Wild V, Jaff D, Shah NS, Frick M. Tuberculosis, human rights and ethics considerations along the route of a highly vulnerable migrant from sub-Saharan Africa to Europe. Int J Tuberc Lung Dis. 2017 Oct 1;21(10):1075-1085. doi: 10.5588/ijtld.17.0324. PMID: 28911349; PMCID: PMC5793855.	PubMed	22/11/2023	" Article 12 of the International Covenant on Economic, Social, and Cultural Rights [ICESCR]).[15] ICESCR Article 12 establishes the right of everyone to enjoy the highest attainable standard of physical and mental health and tasks States Parties (i.e., governments that have ratified or acceded to the convention) with upholding the right by taking steps to prevent, treat, and control epidemic diseases and by 'creating conditions which would ensure to all medical service and medical attention in the event of sickness."
Tackling tuberculosis Local government's public health role	Google	22/11/2023	To reduce the TB burden the TB strategy recommends 10 areas for action including "ensure an appropriate workforce to deliver TB control"
Michael J. Selgelid, Ethics, Tuberculosis and Globalization, *Public Health Ethics*, Volume 1, Issue 1, April 2008, Pages 10–20, https://doi.org/10.1093/phe/phn001	Google	22/11/2023	Discussion of ethics of "Coercive Social Distancing" page 15

Appendix 1:

Search history in databases – conducted on 22/11/2023

PubMed:

Search number	Query	Results
9	((TB OR tuberculosis) AND (expert panel OR advisory panel)) AND (management)	52
5	((public health) AND (advisory panel)) AND (chronic infection)	72
8	"public health advisory panel" OR "independent public health advisory panel"	4,349
7	("public health advisory board" OR "public health advisory panel" OR "independent advisory panel") AND ("chronic infection")	28
6	(((public health advisory board) OR (public health advisory panel))) AND (chronic tuberculosis OR chronic TB)	100
4	(public health) AND (advisory board)	36,897
3	((public health) AND (advis* OR advice OR panel OR consult* OR board)) AND (infectious disease OR infection OR chronic infection)	87,891
2	((advisor* board OR advisory OR consult* board OR consult* OR committee OR panel OR board OR expert*) AND (tuberculosis OR TB OR consumption OR phthisis OR XDR-TB OR extensively drug-resistant tuberculosis OR XDRTB OR extensively drug resistant tuberculosis)) AND (public health)	73,129
1	((advisor* board OR advisors OR consult* board OR consult* OR committee OR panel OR board OR expert*) AND (tuberculosis OR TB OR consumption OR phithisis OR XDR-TB OR extensively drug-resistant tuberculosis OR XDRTB OR extensively drug resistant tuberculosis)) AND (public health)	72,448

Ovid:

- (public health OR community health) AND (advis* board OR advis* panel OR expert OR panel OR committee) AND (tuberculosis OR TB OR consumption OR phthisis)

- Search found 4 books none of which were relevant to the question posed

Google Scholar:

- (public health OR community health) AND (advis* board OR advis* panel OR expert OR panel OR committee) AND (tuberculosis OR TB OR consumption OR phthisis)
    - Search found 26/700 results, the top 30 were reviewed and none were relevant to the question posed

Google:

- (public health OR community health) AND (advis* board OR advis* panel OR expert OR panel OR committee) AND (tuberculosis OR TB OR consumption OR phthisis)
