# Supplementary material for: A decision-making model for public health authorities in circumstances of potentially high public risk
Source: J Public Health (Oxf). 2025 May 18;47(3):550–7. doi: 10.1093/pubmed/fdaf052 (PMC12395956; doi:10.1093/pubmed/fdaf052)
Supplement: Supplementary_Data_2_XDRTB_Expert_Multidisciplinary_Panel-Terms_of_Reference_(1)_fdaf052 [file supplementary_data_2_xdrtb_expert_multidisciplinary_panel-terms_of_reference_(1)_fdaf052.pdf]

Multidisciplinary Expert Public Health Advisory Panel- Extensively Drug Resistant Tuberculosis case

17/01/2024

Purpose and Background:

A multidisciplinary expert panel ("the Panel") has been requested by the multiagency incident management team (IMT) for a complex Extensively Drug Resistant Tuberculosis (XDRTB) case to provide advice to those bodies responsible for the provision and delivery of health and social care, and to recommend the appropriate and proportionate public health action to balance the interest and rights of the individual with the need to reduce the risk of transmission/ exposure of others to XDRTB.

The IMT has asked for an Advisory Panel to assist in this complex case because the multiple factors relating to the interests of the individual and of the wider community are, apparently, in conflict with one another.

The Panel will convene experts in a range of disciplines relevant to the situational context, including experts in bioethics, public health law, patient advocacy, as well as clinical and public health subject matter experts.

Terms of Reference (based on the Code of Practice for Scientific Advisory Committees (CoPSAC 2021. Government Office for Science, UK Government):

The Panel Will:

1) Act independently, agree to participate in the discussions and review information provided by the IMT to understand the case, context, current treatment and management, arrangements, and future risks and concerns

2) Consider the risk and impacts for the individual, the wider population and for the public's health, in the best, current, and reasonable worst-case scenarios

3) Consider the options for management from relevant perspectives including from a legal, ethical, clinical, public health and individual's perspective. Commissioning considerations are out of scope for this panel.

4) Provide objective expert input to the best of their knowledge and ability, to produce recommendations to the IMT on:

   a) The preferred clinical and public health interventions to mitigate the risk and impacts identified
   b) Monitoring the clinical and public health effectiveness of interventions
   c) The ongoing involvement of individuals and communications with them

5) To make any relevant recommendations that may assist the IMT in their ongoing work

6) Conduct a debrief/and or an evaluation after the conclusion of the Panel and contribute to learning in this area

The panel will meet for XDRTB case (Jan 2024- Feb 2024)

1) **First meeting** of the Panel will be focused on:
   a) Terms of Reference, purpose, accountability, and governance of the Panel.
   b) Review of the background information and consideration of the questions put to the Panel
   c) Identification of any additional questions that the Panel will consider.

2) **Second meeting** of the Panel will further consider the identified questions and will identify and review the options to mitigate, risk and impacts.

3) **Third meeting** of the Panel will focus on the recommendations and the rationale of this advice to the IMT and will result in an output for the IMT

4) **Debrief session/ After Action review** with subsequent preparation of a learning report to inform future use of similar Panels.

Risk and Issues

- Success will be predicated on participation of a quorum of appropriate expert members.
- Speed and timeliness of convening of panel and advice can be an issue in this case.
- Little precedence within UKHSA in how expert panels are established, convened and work in case management.

Assumptions/ Dependencies

- Operational aspects of recommendations from the Panel may not be achievable or easy to deliver by the IMT

- The panel will provide their services for no additional fee (all Panel meetings are expected to take place online by Microsoft Teams and therefore Panel members are unlikely to incur any travel costs)

- The panel will declare any conflicts of interests (personal or professional) in relation to any item under discussion at the Panel meeting. If a conflict is declared, then that member may be excluded from discussions, at the discretion of the Chair. Any member that fails to declare a conflict, which subsequently becomes evident, may be expelled from the Panel

- Confidentiality: each panel member must verbally adhere to the following principals of NHS Confidentiality Policy:

1) Person-identifiable or confidential information must be effectively protected against improper disclosure when it is received, stored, transmitted or disposed of.

2) Access to person-identifiable or confidential information must be on a need-to know basis.

3) Disclosure of person identifiable or confidential information must be limited to that purpose for which it is required.

4) Recipients of disclosed information must respect that it is given to them in confidence.

5) If the decision is taken to disclose information, that decision must be justified and documented.

Membership and secretariat - rationale of each role on panel

**Chair**	[redacted]
**Core members and areas represented**	**Ethics** [redacted]<br>**Legal** ([redacted]<br>**Experts by experience** [redacted]<br>**Subject matter expert – Clinical and Public Health** [redacted]<br>**Local Authority/DPH** [redacted]<br>**IMT representation** [redacted]<br>**Evaluation and Associate Caldicott Guardian** [redacted]<br>**Communication** [redacted]<br>**Observers** [redacted]
**Organisation and co-ordination**	[redacted]

**Accountability**	[redacted]
**Meetings**	First meeting 17th January 2024 and two follow up meetings
**Evaluation**	Post evaluation of this initial Panel (tbc)<br>After action review +/- Academic review

Indemnity

From the date that a Panel member agrees to be a member of the Panel, UKHSA agrees to indemnify each member in respect of any action or claim which may be brought, or threatened to be brought, against them either individually or collectively by reason of or in connection with the performance at any time of their duties as Panel members, against all liabilities, costs, expenses, damages and losses (but excluding any indirect or consequential losses) suffered or incurred by a Panel member to the extent that such liability, cost, expense, damages or loss could not have reasonably be covered by the Panel member's existing insurance policies.
