# Supplementary material for: A decision-making model for public health authorities in circumstances of potentially high public risk
Source: J Public Health (Oxf). 2025 May 18;47(3):550–7. doi: 10.1093/pubmed/fdaf052 (PMC12395956; doi:10.1093/pubmed/fdaf052)
Supplement: Supplementary_Data_3_XDRTB_Expert_Background_Info_fdaf052 [file supplementary_data_3_xdrtb_expert_background_info_fdaf052.pdf]

XDRTB Expert Multidisciplinary Panel – Public Consultation Group Advertisement

How much risk of transmission do you find acceptable?

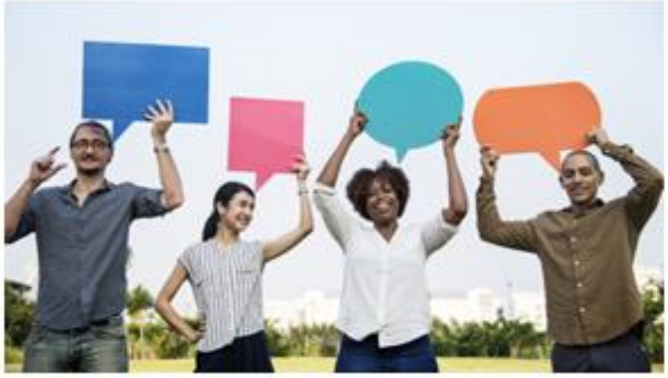

We have been asked by our colleagues at the UK Health Security Agency (UKHSA) to support them with understanding the public's view on what is considered an acceptable level of risk of transmission for a case scenario.

Your opinions will play a role in guiding the decisions of an expert panel on how to handle this case.

We would like to hear from you if you are interested in this opportunity. You do not need to have any specific experiences to contribute.

You will be paid £25 per hour for your time.

If you are interested in this opportunity, please contact:

[redacted]

XDRTB Expert Multidisciplinary Panel – Letter to Public Consultation Participants

Good morning all,

We have been asked by our colleagues at the UK Health Security Agency (UKHSA) to support them with understanding the public's view on what is considered an acceptable level of risk of transmission for a case scenario. The UKHSA safeguards the population from infectious diseases, chemical, biological, radiological, and nuclear incidents, and other health threats. It offers expertise and leadership nationally, locally, and globally to ensure the nation's health security.

What will be discussed?

Our colleagues at the UK Health Security Agency (UKHSA) are inviting you to participate in a discussion regarding a case. Currently, there is an expert panel of individuals offering legal and ethical advice to guide the management team in making optimal case management decisions. They want to incorporate public perspectives into these expert panel discussions and decision-making processes. No specific experience is required and during the meeting various questions and scenarios will be presented to you, which you will be asked to discuss.

Will the meeting be recorded?

Yes, we would like to record the meeting to make anonymised notes which will be shared with UKHSA colleagues and the expert panel. Extracts from the recording may be used as quotes to illustrate key topics discussed. If you would like to join this meeting, we will ask you to complete a permission form.

When is the meeting?

Due to the nature of this work, we have been asked to meet as soon as possible.

Please could you let me know which dates from the following two options would work for you? We will select the date that most people can attend.

Tuesday 16th January: 5pm-6.30pm
Thursday 18th January: 5.30pm-7pm

Where is the meeting?

The meeting will be online (zoom).

How long is the meeting and will I be paid?

The meeting will last for 1.5 hours, and you will be paid at the NIHR payment rate of £25 per hour.

If you have any questions, please do get in touch.

Best wishes,

XDRTB Expert Multidisciplinary Panel – Public Consultation Group Background Information

Adapted from TBalert.org

What is Tuberculosis (TB)?

- TB is an infection caused by a bacterium, *Mycobacterium tuberculosis*.
- TB bacteria are released into the air when someone with infectious TB coughs or sneezes.
  - Pulmonary TB (in the lungs or throat) is the only form of the illness that is infectious, but TB can affect any part of the body.
- If left untreated, TB is a life-threatening illness.
- Most people who breathe in TB bacteria do not become unwell. Their immune systems are strong enough to clear TB completely, or hold it in a latent (sleeping) state. Latent TB can later become active, making someone ill, if their immune system is weakened. A person with latent TB cannot pass TB on to others.

Who is at risk from TB?

- People are most at risk if they know someone who already has, or has had, TB as it usually takes eight hours or more exposure for enough TB bacteria to build up in the body to be a risk.
- Anyone can be affected by TB, but there are certain risk factors:
  - Living where TB is common eg urban areas
  - Homelessness, or living in poorly ventilated or overcrowded accommodation
  - Compromised immune system eg poor nutrition, poverty, poor housing and substance misuse
  - Other illnesses eg people living with HIV
- A person with untreated infectious TB passes the illness on to 10-15 other people, on average, each year without knowing.

What is the treatment for TB?

- Antibiotic treatment cures nearly 90% of cases of standard TB and 48% of cases of drug-resistant TB worldwide.
- However, treatment is not quick or easy:
  - Standard treatment is with a mixture of 4 antibiotics and lasts at least 6 months
  - Individuals may begin to feel better within two weeks of beginning treatment, but it is vital treatment is completed, so that the TB bacteria are completely killed off in the body. This prevents symptoms from returning and the risk of bacteria becoming drug resistant.

- Common side effects to the treatment are feeling sick or dizzy, skin rashes, pins and needles and flu-like symptoms. Rarer side effects include jaundice (yellowing of the skin or eyes).
- Drug resistant TB requires a longer course of treatment with different combinations of medications that can have further side effects. Duration of treatment can also be longer and exact course of treatment is dependent on the case. Some cases require surgery to remove part of the lung.
- Directly Observed Treatment is recommended for people who have difficulty keeping to a course of treatment – perhaps through lifestyle factors such as homelessness. It involves someone eg TB nurses trained volunteers meeting regularly with the individual to watch them take their medication. This ensures that the right medication is taken in the right doses, at the right time, for as long as it's required.

What is drug resistant TB?

- Multi drug-resistant TB (MDR-TB) and extensively drug-resistant TB (XDRTB) are big global health threats. The World Health Organisation estimates that 480,000 people developed MDR-TB in 2015, but only 52% of cases were identified and treated appropriately.
- Drug-resistant forms of TB can develop if treatment is incorrect or incomplete. People with infectious drug-resistant TB can then also pass this drug-resistant strain on to others.
- The standard treatment is not effective. Treatment is with drugs that are less effective, more toxic and much more costly. Treatment can take up to two years, cure rates are lower and fatalities are higher.
- Treatment is even more complicated for people with XDR-TB, and it is estimated that globally around 70% of patients die within a month of diagnosis.
