# Supplementary material for: A decision-making model for public health authorities in circumstances of potentially high public risk
Source: J Public Health (Oxf). 2025 May 18;47(3):550–7. doi: 10.1093/pubmed/fdaf052 (PMC12395956; doi:10.1093/pubmed/fdaf052)
Supplement: Supplementary_Data_4_XDRTB_Expert_Multidisciplinary_TB_Evidence_Summary_fdaf052 [file supplementary_data_4_xdrtb_expert_multidisciplinary_tb_evidence_summary_fdaf052.pdf]

Surgery to treat MDR/XDR pulmonary TB

**First author**	Marrone	Chiang	Kang	Naidoo	Park	Papiashivili	Dravniece	Shiraishi	Idriss	Vashakidze	Fox
**Study date**	1975-2012	1990-1997	1996-2008	1997-2005	1994-2004	1998-2011	1999-2005	2000-2007	2007-2010	2008-2012	1994-2008
**Study type**	Syst review	Retro cohort (RC)	RC	RC	Prospective cohort	RC	RC	RC	RC	RC	IPD analysis
**Location**	Global	Taiwan	South Korea	South Africa	Korea	Israel	Latvia	Japan	South Africa	Georgia	Global
**Disease type**	MDR, XDR	MDR	MDR, XDR	MDR	MDR, XDR	MDR	XDR	MDR	XDR	MDR, XDR	MDR
**Number**	1572	27	72	27	19	17	17	56	4	109	478
**Surgery**		48% lobectomy	53% lobectormy	37% lobectomy	74% lobectomy	21% lobectomy	24% lobectomy	54% lobectomy	50% lobectomy	47% lobectomy	>50% lobectomy
**Additional drug Rx**	None in single arm	Yes	Yes – pre & post surgery	Yes – pre & post surgery	Yes – though poor drugs post surgery	Yes	Yes – pre and post	Yes	Yes	Yes	Yes
**Outcome (incl definition)**	Laserson outcomes. From 23 single arm studies, successful Rx 12 months from surgery alone 87% (95%CI 83-91)	26 patients alive post surgery. Mean 15/12 of Rx post surgery. 23 completed Rx at follow up, 85% SRx at mean 42/12 from end of Rx	Laserson outcomes. SRx 90% overall (85% XDR, 93% MDR)	93% "cure" – though definition not clear	79% "cure" ie no relapse at mean of 53 months	Laserson outcomes. 71% SRx	Laserson outcomes. 47% SRx	Relapse in post-surgery smear in 5/56 ie "cure" in 92% at end of Rx	Laserson outcomes. 50% Cure, 50% TC	Laserson outcomes (though not referenced). SRx 76%	Laserson outcomes. 70% SRx
**Comment**	73 XDR – 69% (27-93) ie less than for MDR at 12/12		26 (36%) had XDR		2 XDR				Most patients referred had bilat disease and so didn't have surgery	28% XDR	10% XDR, 35% FQN & injectable Resistance unknown. Better outcome with more drugs. Better outcome with limited surgery (ie lobectomy vs pneumonectomy). Better outcome

											with sputum conversion pre-surgery
**Ref**	IJTLD 2013;17:6	IJTLD 2-11;5:272	Ann Thoracic Surg 2010;89:1597	Asian Cardiovasc Thorac Ann 2007;15:134	Int J Infect Dis 2009;13:170	Israel Med Assoc J 2012;14: 733	ERJ 2009;34:180	J Thorac Cardiovasc Surg 2008;138:1150	Ann Thorac Surg 2012;94:381	Eur J Cardiothorac Surg 2021;60:1279	CID 2016;92:887

<u>Definitions (from Laserson IJTLD 2005;9:640). Successful Rx (SRx) = C + TC below</u>

Cure (C) - An MDR-TB patient who has completed treatment according to country protocol and has been consistently culture-negative (with at least five results) for the final 12 months of treatment. If only one positive culture† is reported during that time, and there is no concomitant clinical evidence of deterioration, a patient may still be considered cured, provided that this positive culture is followed by a minimum of three consecutive negative cultures, taken at least 30 days apart.

Treatment completed (TC) - An MDR-TB patient who has completed treatment according to country protocol but does not meet the definition for cure or treatment failure due to lack of bacteriologic results (i.e., fewer than five cultures were performed in the final 12 months of therapy).

Treatment failure - Treatment will be considered to have failed if two or more of the five cultures recorded in the final 12 months are positive, or if any one of the final three cultures is positive. Treatment will also be considered to have failed if a clinical decision has been made to terminate treatment early due to poor response or adverse events.
