# Supplementary material for: A decision-making model for public health authorities in circumstances of potentially high public risk
Source: J Public Health (Oxf). 2025 May 18;47(3):550–7. doi: 10.1093/pubmed/fdaf052 (PMC12395956; doi:10.1093/pubmed/fdaf052)
Supplement: Supplementary_Data_5_Expert_Multidisciplinary_Panel-Template_Risk_Assessment_Matrix_fdaf052 [file supplementary_data_5_expert_multidisciplinary_panel-template_risk_assessment_matrix_fdaf052.pdf]

Scenario	Probability of Continuing Disease	Probability of Transmission	Impact on Individual Infected	Impact on Public	Risk to Individual (use table 1)	Risk * to Public (use table 2)	Target Risk of Transmission to Individual (acceptable risk)	Target Risk of Transmission to Public (Outbreaks) (acceptable risk)	Interventions to Reduce Transmission	Residual Risk of Transmission to an Individual	Residual Risk of Transmission to Public (Outbreaks)	Rationale and Assumptions
**Infectious and non-compliant**												
**Infectious and compliant**												
**Likely non-infectious and non-compliant up to 2 years**												
**Likely non-infectious and non-compliant after 2 years**												
**Non-infectious and compliant up to 2 years**												
**Non-infectious and compliant after 2 years**												
**Patient lost to follow up and infection status unknown up to 2 years**												
**Patient lost to follow up and infection status unknown after 2 years**												

Table 1: Calculating risk to an Individual

	**Probability of Transmission**					
**Impact on Individual Infected**	Very High	High	Moderate	Low	Very Low	None
Very High	Extreme risk	Extreme risk	Very high risk	High risk	Medium risk	No risk
High	Extreme risk of transmission	Very high risk of transmission	High risk of transmission	Medium risk of transmission	Medium risk of transmission	No risk of transmission
Moderate	Very high risk of transmission	High risk of transmission	Medium risk of transmission	Medium risk of transmission	Low risk of transmission	No risk of transmission
Low	High risk of transmission	Medium risk of transmission	Medium risk of transmission	Low risk of transmission	Very low risk of transmission	No risk of transmission
Very Low	Medium risk of transmission	Medium risk of transmission	Low risk of transmission	Very low risk of transmission	Very low risk of transmission	No risk of transmission

Risk = probability of transmission x impact

Table 2: Calculating risk to the Public

	**Probability of Transmission**					
**Impact on the Public**	Very High	High	Moderate	Low	Very Low	None
Very High	Extreme risk of transmission	Extreme risk of transmission	Very high risk of transmission	High risk of transmission	Medium risk of transmission	No risk of transmission
High	Extreme risk of transmission	Very high risk of transmission	High risk of transmission	Medium risk of transmission	Medium risk of transmission	No risk of transmission
Moderate	Very high risk of transmission	High risk of transmission	Medium risk of transmission	Medium risk of transmission	Low risk of transmission	No risk of transmission
Low	High risk of transmission	Medium risk of transmission	Medium risk of transmission	Low risk of transmission	Very low risk of transmission	No risk of transmission
Very Low	Medium risk of transmission	Medium risk of transmission	Low risk of transmission	Very low risk of transmission	Very low risk of transmission	No risk of transmission
